# Increased alloreactive and autoreactive antihuman leucocyte antigen antibodies associated with systemic lupus erythematosus and rheumatoid arthritis

**DOI:** 10.1136/lupus-2018-000278

**Published:** 2018-09-25

**Authors:** Rachael P Jackman, Giovanna I Cruz, Joanne Nititham, Darrell J Triulzi, Lisa F Barcellos, Lindsey A Criswell, Philip J Norris, Michael P Busch

**Affiliations:** 1 Blood Systems Research Institute, San Francisco, California, USA; 2 Department of Laboratory Medicine, University of California, San Francisco, California, USA; 3 School of Public Health, University of California, Berkeley, California, USA; 4 Russell/Engleman Rheumatology Research Center, Department of Medicine, University of California, San Francisco, California, USA; 5 Institute for Transfusion Medicine, University of Pittsburgh, Pittsburgh, Pennsylvania, USA

**Keywords:** human leukocyte antigen (HLA), alloantibodies, autoantibodies, rheumatoid arthritis (RA), systemic lupus erythematosus (SLE)

## Abstract

**Objectives:**

Rheumatoid arthritis (RA) and systemic lupus erythematosus (SLE) disproportionately affect women during and following childbearing years. Antihuman leucocyte antigen (HLA) alloantibody responses are common in healthy parous women, and as these diseases are both linked with HLA and immune dysregulation, we sought to evaluate anti-HLA antibodies in RA and SLE.

**Methods:**

Anti-HLA antibodies were measured among parous SLE cases (n=224), parous RA cases (n=202) and healthy parous controls (n=239) and compared with each other as well as with nulliparous female and male controls. Antibody specificities were identified and compared against subject HLA types to determine autoreactivity versus alloreactivity. The association of anti-HLA antibodies with clinical outcomes was evaluated.

**Results:**

Levels and frequencies of anti-HLA antibodies were significantly higher among parous females with SLE (52%) or RA (46%) compared with controls (26%), and anti-HLA antibodies were also found among nulliparous females and males with SLE and RA. Autoreactive anti-HLA antibodies were observed among SLE and RA antibody-positive subjects, but not healthy controls, with the highest frequency of autoreactive anti-HLA antibodies found in the SLE subjects. Higher levels of anti-HLA antibodies were associated with nephritis among the nulliparous SLE cases (p<0.01). The presence of anti-class I HLA antibodies was associated with younger age at diagnosis among both the RA and SLE nulliparous cases.

**Conclusions:**

Both autoreactive and alloreactive anti-HLA antibodies were found at high levels in RA and SLE subjects. These occurred even in the absence of alloexposure, particularly among SLE subjects and may be linked with disease severity.

Human leucocyte antigens (HLA) are potent alloantigens, and anti-HLA antibody responses are common following alloexposures such as solid organ transplant, transfusion or pregnancy. In transplant or transfusion recipients, host-generated alloantibodies can contribute to transplant rejection or refractoriness to platelet transfusion.^1–4^ In addition, donor-derived anti-HLA antibodies in blood products are thought to increase the risk of transfusion-related acute lung injury, a major cause of transfusion-related mortality.^5^

For pregnant women, these alloantibodies are generally considered benign, though some studies have shown associations with recurrent miscarriage and preterm labour.^6–8^ The frequency of detected anti-HLA antibodies among parous women is high ~24% and incrementally increases with increasing parity.^9^ These antibodies can appear early in pregnancy and can be quite persistent with antibodies detected decades after exposure.^9–11^

Specific HLA alleles have been associated with several autoimmune diseases, including rheumatoid arthritis (RA) and systemic lupus erythematosus (SLE). DRB1*0301 and DRB1*1501 are associated with increased risk of SLE, with the former specifically linked to anti-La and anti-Ro antibody positive cases and the latter to anti-Sm antibodies.^12–14^ The ‘shared epitope’ encoded by several DRB1 alleles is strongly linked to RA development and disease severity,^15^ as are polymorphisms in the DPB1 and B loci.^16^

Pregnancy can also have important implications for both SLE and RA. Women are at much higher risk of developing these diseases, particularly during and following their childbearing years, with peak incidence of SLE among young women and incidence of RA peaking later in life.^17 18^ The risk of a new RA diagnosis is increased in the postpartum period.^19 20^ This may in part be explained by exposure to cells of fetal origin, as mothers whose children carry high-risk HLA alleles have an increased risk of both RA and SLE,^21 22^ and microchimeric populations of cells carrying DRB1 alleles with the shared epitope have been found at a higher frequency in RA cases versus controls.^23^ For those with existing RA, however, pregnancy frequently results in temporary remission,^24^ with greater class II HLA disparity between mother and child associated with increased likelihood of RA remission during pregnancy.^25^

Given the importance of the HLA region to SLE and RA, the prevalence of these diseases among women, and the immune dysregulation associated with SLE and RA, we evaluated the frequency of anti-HLA antibodies among parous patients with RA and SLE compared with healthy controls with similar alloexposures. We also identified the specificities of these anti-HLA antibodies to determine whether any of them were autoreactive and looked for associations of these anti-HLA antibodies with clinical outcomes. We hypothesised that higher frequencies of HLA antibodies would be found among parous women with SLE and RA than healthy parous controls and that these antibodies would be linked with more severe clinical outcomes.

## Methods

### Subjects

Parous female SLE and RA cases were drawn from the University of California San Francisco (UCSF) Mother-Child Immunogenetic Study.^21^ ^22^ Nulliparous female and male SLE cases were selected from the UCSF Lupus Genetics Project Collection.^21^ Nulliparous female and male RA cases were selected from the UCSF Rheumatoid Arthritis Genetics Project.^21 22^ Medical record reviews confirmed that all cases met the respective classification criteria for RA and SLE as established by the American College of Rheumatology.^26 27^ Healthy controls were obtained from the Leucocyte Antibody Prevalence Study, with the full cohort used for males and nulliparous females, and a subset of recalled donors used for the parous females.^9^ Table 1 outlines the number of subjects per group and details of the number of subjects included in each analysis. The large majority of subjects were Caucasian, however, 13% of the nulliparous female controls, 10% of the male controls, 7% of the parous female controls and <1% of the SLE parous females were not Caucasian. All subjects provided informed written consent.

**Table 1:** Subjects included by analysis

	Screened for human leucocyte antigen Abs	Screened for Class I Ag specificities	Screened for Class II Ag specificities	Age of diagnosis analysis	Nephritis analysis	Erosion analysis
Healthy						
Nulliparous males	1148	0	0	–	–	–
Nulliparous females	1757	0	0	–	–	–
Parous females	239	35*	42*	–	–	–
Systemic lupus erythematosus						
Nulliparous males	48	9*	15*	48	48	–
Nulliparous females	48	5*	15*	47†	48	–
Parous females	224	25	25	224	223‡	–
Rheumatoid arthritis						
Nulliparous males	48	0	0	39†	–	39§
Nulliparous females	48	0	0	48	–	31§
Parous females	202	25	25	184†	–	161§

*All antibody positive samples screened.

†Age of diagnosis not available on some subjects.

‡Data on nephritis missing for one subject.

§Data on erosions missing for some subjects.

### Anti-HLA antibody screening

Overall, antibodies against class I and class II HLA antigens were measured using the One Lambda LabScreen Mixed Luminex assay (Canoga Park, California, USA), a bead-based multiplexing system. Serum or plasma samples were screened according to the manufacturer’s instructions. This kit uses multiantigen coated beads and reports results as a normalised to background (NBG) ratio for each bead. We used the highest reported value for each sample. Cut-off values for positive antibody results were set as previously described to be three SD above the mean value measured in a large panel of unexposed healthy subjects, with a NBG ratio greater than 10.8 for class I or 6.9 for class II considered antibody positive.^9 28^

Antibody specificities were evaluated using the One Lambda LabScreen Single Antigen HLA class I and class II assays and analysed using HLA Fusion V.3.0 (One Lambda).

### Identification of autoreactive anti-HLA antibodies

Subject HLA types were imputed at two-field resolution as previously described.^21^ Alleles with a low level of imputation accuracy (r^2^<0.3) were excluded. Previous work comparing typed and imputed DRB1 demonstrated that individually, ≥98% of alleles were accurately called. To validate the current imputation data, imputed DRB1 types were compared against direct genotyping results for 47 of the parous female SLE and RA subjects (94 alleles) who were screened for antibody specificity. An exact match at the genotype level was found for 93%, with the remaining 7% remaining accurate at the one-field level of resolution. None of the errors in the imputed DRB1 types resulted in any changes to the auto-determinations versus allo-determinations for typed antibodies. Specificities of anti-HLA antibodies were compared against imputed subject HLA types to identify any autoreactive specificities. Individuals were considered positive for autoreactive anti-HLA antibodies if one or more of their specificities were against their own class I or class II HLA alleles. Three increasingly stringent cut-offs were evaluated for the antibody specificity assay with X2 the least stringent and X6 the most stringent. The manufacturer established these proprietary cut-offs, so we do not have specific details on how they are calculated. As we did not have DPA1 types on most subjects, antibody specificities against potential DP autoantigens were not included in this analysis.

### Statistical analysis

Prism (GraphPad Software, La Jolla, California, USA) was used for all statistical analysis. Comparisons of antibody levels between multiple groups were made with one-way analysis of variance (ANOVA) using Dunnett’s post-test to compare each case group to healthy controls. Comparisons of frequencies were made by χ^2^ test. Comparisons of mean values between two groups were done by unpaired t-test. Differences were reported as significant if p<0.05.

## Results

Anti-HLA antibodies can be found in many healthy females as the result of previous pregnancies, but only rarely in males or in nulliparous females.^9^ We evaluated the frequency of these antibodies in parous females with SLE and RA to see if they could be found with greater frequency in these populations compared with healthy parous females. Our cohorts of parous females had similar distributions of number of pregnancies among the healthy, SLE and RA subjects, though RA subjects appeared to be slightly skewed towards fewer pregnancies (online supplementary figure 1). A comparison of mean number of pregnancies between groups by ANOVA showed no significant difference between either the SLE or RA groups and the controls.

10.1136/lupus-2018-000278.supp1 Supplementary data

**Figure 1:**
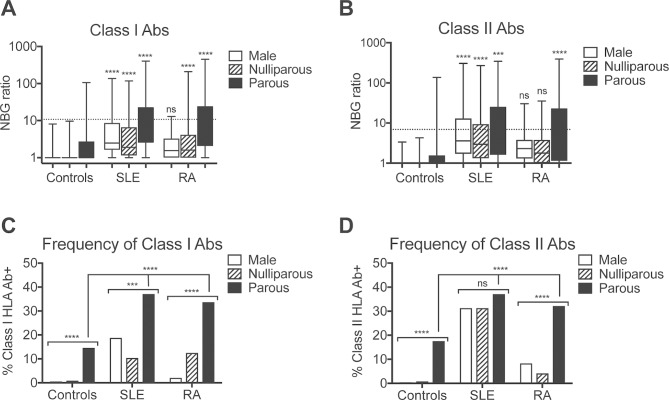
Increased anti-HLA antibodies among SLE and RA subjects. (A) Class I and (B) class II anti-HLA antibody NBG ratios are plotted by group for healthy males (n=1148), healthy nulliparous females (n=1757), healthy parous females (n=239), males with SLE (n=48), nulliparous females with SLE (n=48), parous females with SLE (n=224), males with RA (n=48), nulliparous females with RA (n=48) and parous females with RA (n=202). Dashed lines indicate cut-offs used to identify antibody positive samples. Boxes display IQR with median indicated with a line. Whiskers display fifth to 95th percentiles. SLE, RA and healthy controls were compared by exposure group (eg, males, nulliparous females and parous females) by analysis of variance with Dunnett’s post-test used to compare each to the matched healthy control. (C) The frequency of class I and (D) class II anti-HLA antibodies is plotted by the same groups as above. Frequencies of anti-HLA antibodies were compared between male, nulliparous female and parous female groups among SLE, RA or healthy subjects and between healthy, SLE and RA subjects for the parous females by χ^2^ test. ***P<0.001, ****P<0.0001, ^ns^P>0.05. HLA, human leucocyte antigen; NBG, normalised to background; SLE, systemic lupus erythematosus; RA, rheumatoid arthritis.

The levels (figure 1A,B) and the frequency (figure 1C,D) of both class I and class II anti-HLA antibodies were higher in the SLE and RA groups as compared with the healthy group. Parous women with either SLE or RA were about twice as likely to have these antibodies compared with healthy controls (52% of SLE and 46% of RA, compared with 26% of healthy controls had anti-class I and/or anticlass II HLA antibodies). In addition, when we evaluated males and nulliparous females for anti-HLA antibodies, we found that while the frequency of these antibodies was generally lower than what is seen in parous females with SLE or RA, they could be found among the SLE and RA groups. The frequency of anti-class II HLA antibodies was high in all three SLE groups, with no significant difference between parous females, nulliparous females and males.

**Figure 2:**
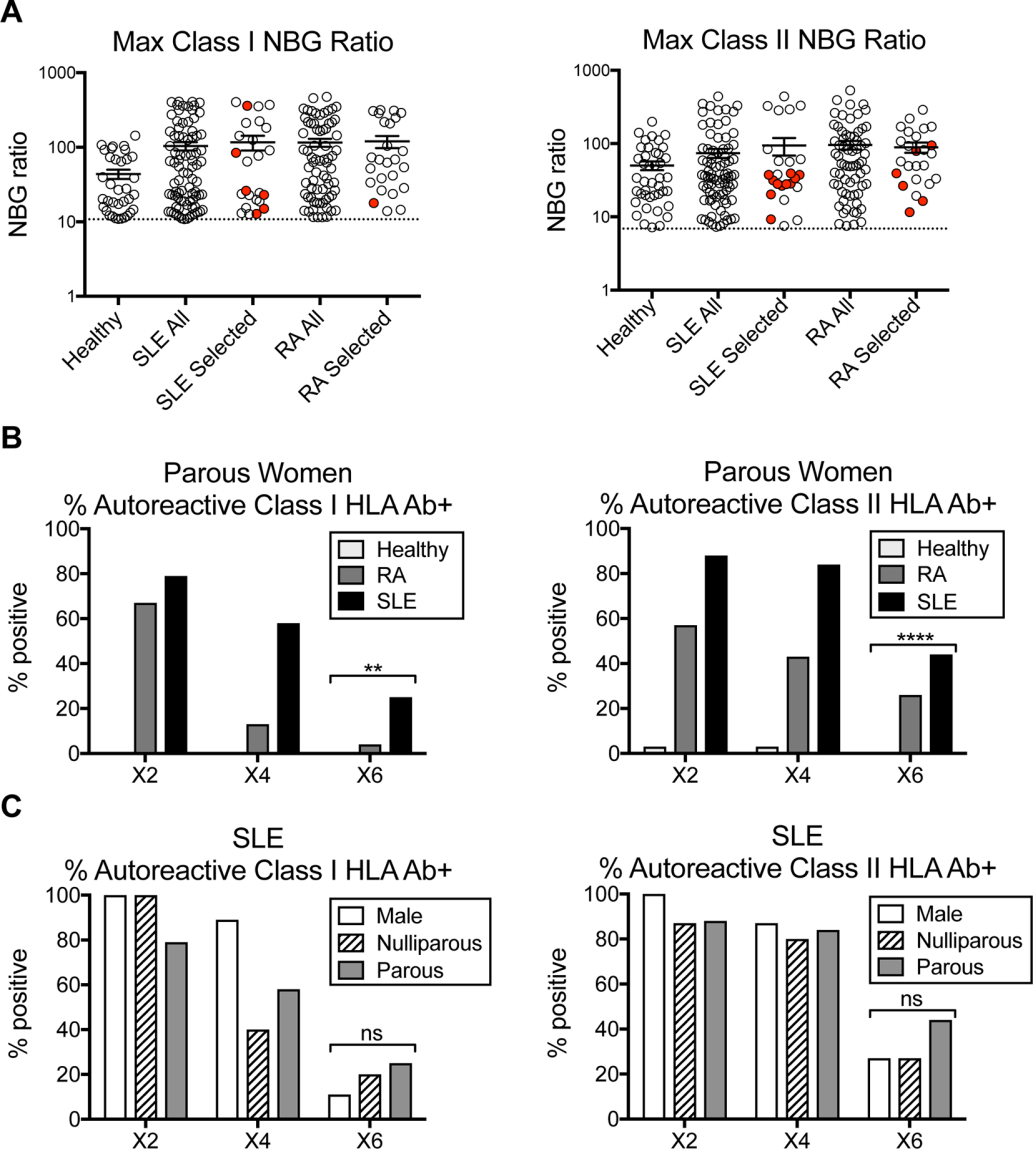
Presence of autoreactive anti-HLA antibodies among SLE and RA subjects. (A) A subset of antibody positive parous female SLE and RA subjects was selected for antibody specificity screening. Selected samples are plotted side by side with their parent populations to compare NBG ratio distributions for class I (left panel) and class II (right panel) anti-HLA antibodies. Samples with autoreactive anti-HLA antibodies are indicated in red. Dashed lines indicate cut-offs used to identify antibody positive samples. (B) The frequency of one or more autoreactive class I (left panel) or class II (right panel) antibodies is plotted for parous female subjects by disease category—healthy, SLE or RA. Three increasingly stringent cut-offs were used to detect particular antibody specificities (X2 is the least stringent and X6 the most stringent). Groups were compared using the X6 cut-off by χ^2^ test. (C) The same analysis was used as in (B) above, this time evaluating the specificities of the male, nulliparous female and parous female SLE subjects. These three groups were compared for the X6 cut-off by χ^2^-test. **P<0.01, ****P<0.0001, ^ns^P>0.05.

**Figure 3:**
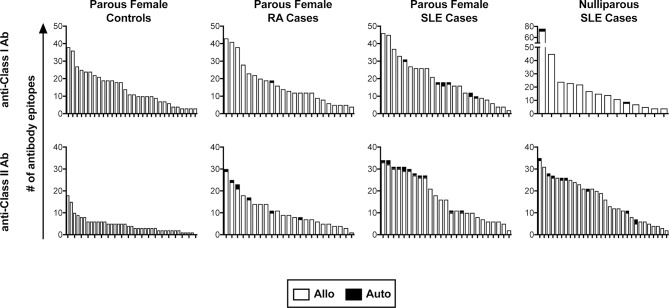
Distribution of autoreactive and alloreactive epitopes of anti-HLA antibodies. The number of anti-class I and anti-class II HLA epitopes detected at the X6 cut-off are plotted by subject with autoreactive epitopes shaded black. Each bar represents the distribution of epitopes for an individual subject. HLA, human leucocyte antigen; SLE, systemic lupus erythematosus; RA, rheumatoid arthritis.

We next evaluated the specificities of these anti-HLA antibodies to determine if any of them were specific for autoantigens. For the parous females, all healthy subjects positive for antibodies were evaluated, while a subset of 25 class I positive and 25 class II positive subjects were selected from among the SLE subjects and from among the RA subjects. Levels of anti-HLA antibodies in selected samples were compared against those from the full pool of positive samples to confirm a representative distribution of antibody levels (figure 2A). Three different cut-offs were evaluated for assessing the presence of a given antibody specificity, the manufacturer’s default (X6) or two others with decreasing stringencies (X4 and X2). Using the X6 cut-off, we found autoreactive antibodies in a subset of the SLE and RA subjects, but not among the healthy controls (figure 2B). We next evaluated the levels of total anti-HLA antibody in those positive for autoreactive antibodies to see if they were associated with higher levels of total antibody, but they were distributed in strength throughout the range of positive samples (figure 2A, autoreactive samples indicated in red). We then evaluated the specificities of the anti-HLA antibodies found in the SLE male and nulliparous female cases. These subjects had similar rates of autoreactivity among the antibody-positive subjects (figure 2C). The specificities of each autoreactive anti-HLA antibody identified are listed by group in online supplementary table 1. We also checked the distribution of autoepitopes versus alloepitopes among all the parous female subjects and the nulliparous SLE subjects (figure 3). The majority of the antibodies were alloreactive, even when autoreactive antibodies are present. Furthermore, the cases, particularly the SLE cases, have much wider ranges of class II antibodies, and this greater diversity appears to be unrelated to alloexposures as both the parous and nulliparous subjects had similar distributions.

10.1136/lupus-2018-000278.supp2 Supplementary data

Finally, we looked to see if the presence of these anti-HLA antibodies was linked with any clinical outcomes. For the SLE cases, we looked at dsDNA antibodies, abnormal (meaning abnormally high) immunoglobulin (Ig)G levels, abnormal IgM levels, age at diagnosis and lupus nephritis. For the RA subjects, we evaluated age at diagnosis, cyclic citrullinated peptide antibodies, extra-articular manifestations, rheumatoid factor and erosions.

For the parous female SLE cases, the number of class II antibody epitopes was significantly higher among those with dsDNA antibodies, and the level of class I antibodies was associated with abnormal IgG levels. These differences were modest and did not appear in the nulliparous group, but they were consistent with the hypothesis that anti-HLA antibodies are more likely to occur alongside other abnormal antibody responses (data not shown).

**Figure 4:**
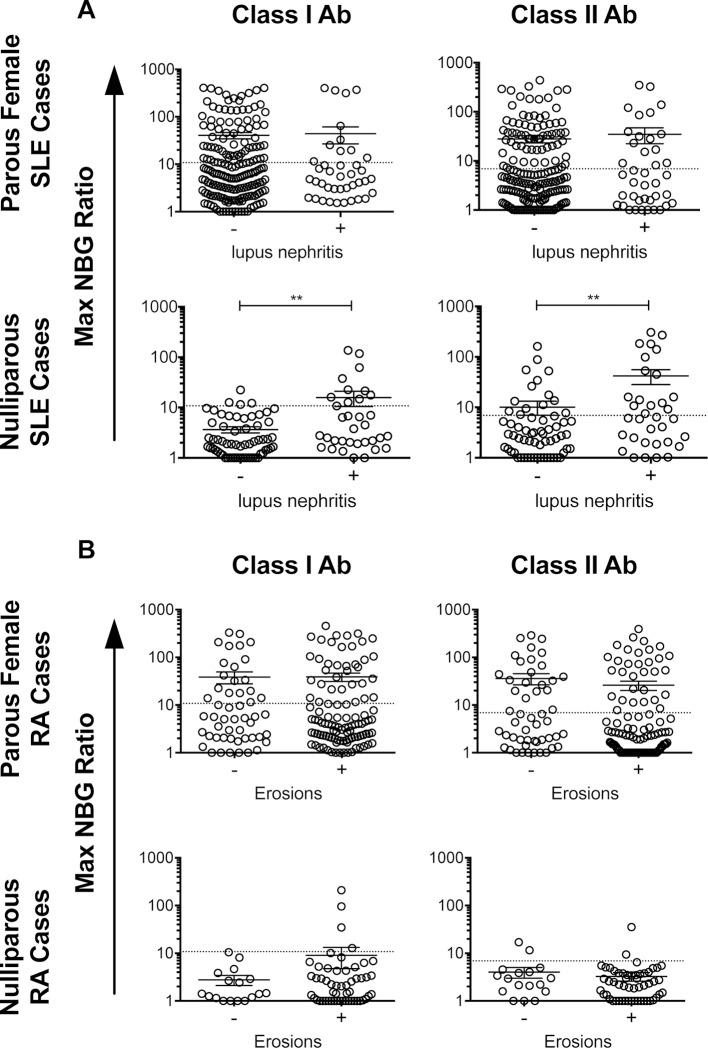
Associations between HLA antibodies and clinical outcomes (A) Plots showing the class I or class II antibody max NBG ratios for parous female and combined nulliparous SLE cases with and without lupus nephritis. (B) Plots showing the class I or class II antibody max NBG ratios for parous female and combined nulliparous RA cases with and without erosions. Dashed lines indicate cut-offs used to identify antibody positive samples.^9^ **P<0.01. HLA, human leucocyte antigen; NBG, normalised to background; SLE, systemic lupus erythematosus; RA, rheumatoid arthritis.

For the nulliparous SLE cases, higher levels of both class I and class II HLA antibodies were associated with lupus nephritis (figure 4A). This was not seen among the parous female cases. No relationship was seen between anti-HLA antibody levels and erosions in the RA cases, though very few of the nulliparous RA cases were positive for anti-HLA antibodies (figure 4B).

**Figure 5:**
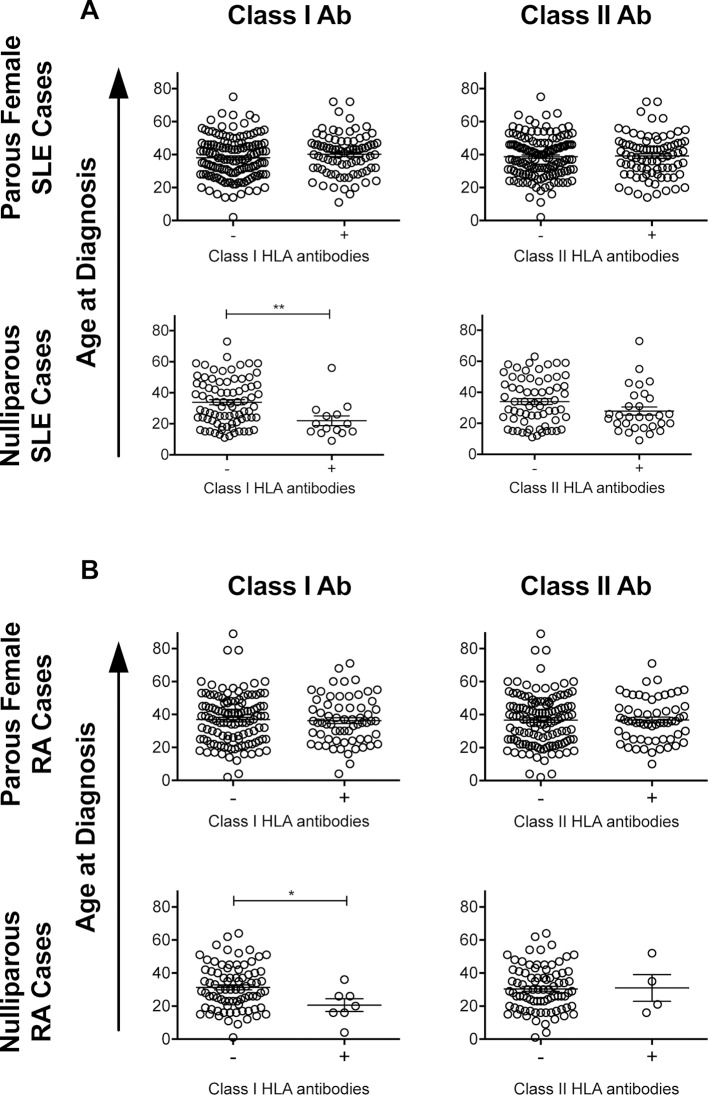
Associations between the HLA antibodies and age at diagnosis. Plots showing age of diagnosis for parous female and combined nulliparous SLE (A) or RA (B) cases with and without class I or class II HLA antibodies. *P <0.05, **P <0.01. HLA, human leucocyte antigen; SLE, systemic lupus erythematosus; RA, rheumatoid arthritis.

Earlier age of diagnosis was associated with the presence of anti-class I HLA antibodies in both the nulliparous SLE (figure 5A) and nulliparous RA (figure 5B) cases. This difference was not seen among parous female cases. There were no significant differences in age of diagnosis associated with the presence of anti-class II HLA antibodies (figure 5).

## Discussion

In this study, we have demonstrated a doubling of the frequency of anti-HLA antibodies among parous women with SLE or RA compared with healthy controls with similar numbers of pregnancies. In addition, anti-HLA antibodies were found in many SLE and some RA subjects without any known alloexposures. While most antibody specificities targeted allogeneic HLA, a significant portion of the SLE and RA subjects carried one or more antibodies against autologous HLA. Increased levels of anti-HLA antibodies were associated with nephritis among nulliparous SLE subjects, and anti-class I HLA antibodies were associated with earlier age of diagnosis for nulliparous SLE and RA subjects.

The higher levels and frequency of antibodies found among parous SLE and RA subjects compared with the healthy parous controls is consistent with the overly sensitive immune system associated with these diseases. This is also reflected in the diversity of antibody specificities, particularly for anti-class II antibody responses, which were far more diverse in the RA and SLE cases compared with controls (figure 3). In addition to hyperactivity of the immune system, the reduced clearance and impaired complement activity associated with SLE could be contributing to the increased alloresponse among these subjects.^29^ Consistent with our findings, there appears to be an increased risk of red cell alloimmunisation following transfusion associated with some autoimmune diseases, including both SLE and RA.^30 31^

While the majority of anti-HLA antibodies were targeting allogeneic HLA antigens, many of the SLE subjects and some of the RA subjects carried one or more antibodies targeting autologous HLA (figure 2). The specificities of these autoreactive antibodies identified by the assay are listed in the online supplementary table 1. One potential limitation is that these bead-based antigen-specific HLA antibody assays can sometimes detect antibodies directed at denatured HLA proteins in addition to those targeting native HLA, due to the presence of some denatured HLA on the beads. These antibodies targeting denatured antigen have been shown to have little relevance in the transplant setting, as they are not associated with increased transplant rejection.^32 33^ While we cannot rule out the possibility that some of our autospecific antibodies are targeting denatured HLA, we think that is unlikely for the following reasons. First, these antidenatured HLA antibodies generally have a low MFI and would likely be eliminated by the more conservative X6 cut-offs used in our analyses.^33^ Second, we do not detect these autoreactive antibodies in our healthy control population, though their presence could be the result of the polyclonal B activation associated with SLE. Finally, particular specificities have been associated with antidenatured HLA antibodies using this assay, and none of our detected autoreactive antibody specificities are on this list (online supplementary figure 1), which are generally more rare HLA types.^33–35^ That said, it is possible that some of the autoreactive (or alloreactive) anti-HLA antibodies we detected could be targeting these less relevant denatured antigens. Interestingly, one study did find a higher prevalence of SLE among kidney transplant candidates who tested positive for these antidenatured HLA antibodies.^34^

While both the SLE and RA cases responded similarly to alloexposure via pregnancy, they differed significantly in the absence of allogeneic exposure. Some anti-HLA antibodies were detected in the nulliparous female and male RA cases, but levels were only significantly higher than controls among nulliparous females, and only for anti-class I antibodies. It is possible that these higher levels of HLA antibodies in nulliparous RA females compared with RA males could be the result of undocumented early miscarriage, but may also represent some other sex effect. In contrast, the nulliparous female and male SLE groups had significantly higher antibodies detected as compared with controls for both class I and class II, and for class II, the frequencies were not significantly different from the alloexposed (parous) SLE group (figure 1). Furthermore, though associations were weak, in the parous female group, the diversity of the anti-class II antibody epitopes was higher among those with anti-dsDNA antibodies, and higher levels of anti-class I was also associated with abnormal IgG levels (data not shown). All of this is consistent with the abnormal B cell receptor signalling and polyclonal activation associated with SLE.^36 37^

The anti-class I and anti-class II antibody responses also differed significantly, particularly among the SLE cases. There were far more antibodies generated against class II antigens for SLE, even in the absence of alloexposure. Furthermore, the frequency of autoreactive antibodies against class II was much higher than those targeting class I, and this is in spite of our inability to include the DP loci in this analysis due to incomplete typing. One possibility is that tolerance mechanisms protecting against anti-class I responses may be more robust than those for class II. This does not seem unreasonable given the ubiquitous expression pattern of class I HLA compared with the more restricted expression of class II. Interestingly, the link seen between age of diagnosis and anti-HLA antibodies was only seen for class I antibodies, even though this association was observed in both the SLE and RA nulliparous groups. This may suggest that the appearance of anti-class I HLA antibodies in the absence of alloexposure may be linked with an increased genetic burden, as this is generally associated with an earlier age of diagnosis.^38^ No link between age of diagnosis and HLA antibodies was seen among the parous female cases, most likely due to the high levels of antibodies seen following pregnancy, which may mask any effect.

It is difficult to assess if these anti-HLA antibodies are contributing to disease or simply an indicator of an imbalanced immune system. We did see a correlation between nephritis and both anti-class I and anti-class II antibodies for the nulliparous SLE cases but were unable to establish a causal relationship. As with age of diagnosis, differences were not observed in the parous female group, again, presumably due to the higher levels of antibodies seen following pregnancy. In addition, both SLE and anti-HLA antibodies have been linked to increased risk of pregnancy loss or preterm labour.^6–8 39–41^ As anti-HLA antibodies were particularly high in the SLE subjects, even in the absence of previous alloexposure, it seems plausible that these antibodies could contribute to pregnancy complications associated with SLE. We did have self-reported data on miscarriage and stillbirth, but did not observe any significant differences in either overall anti-HLA antibody levels or the presence of autoreactive anti-HLA antibodies among SLE subjects with any or >1 miscarriage/stillbirth versus none (data not shown).

In summary, we believe that this study is the first to show an increase in antibodies against HLA associated with SLE and RA. We have found that these antibodies occur more frequently and at higher levels in parous SLE and RA women compared with parous controls with similar exposures. Furthermore, these antibodies occur in the absence of allogeneic exposure, particularly in SLE. While the majority of the antibodies generated are against non-self antigens, we found many of the SLE and some of the RA subjects tested positive for autoreactive anti-HLA antibodies. Further work will be required to determine what role these antibodies play in disease pathogenesis.
