# Supplementary material for: Increased alloreactive and autoreactive antihuman leucocyte antigen antibodies associated with systemic lupus erythematosus and rheumatoid arthritis
Source: Lupus Sci Med. 2018 Sep 25;5(1):e000278. doi: 10.1136/lupus-2018-000278 (PMC6173266; doi:10.1136/lupus-2018-000278)
Supplement: Supplementary data [file lupus-2018-000278supp001.docx]

**Figure S1. Similar numbers of pregnancies among parous female subjects.** The percent of subjects with 1, 2, 3, 4, 5, or ≥6 pregnancies is plotted by group: healthy controls, SLE subjects, and RA subjects. Mean number of pregnancies was compared between groups using ANOVA with Dunnett’s post-test to compared each case group to the healthy controls.
