# Supplementary material for: Increased alloreactive and autoreactive antihuman leucocyte antigen antibodies associated with systemic lupus erythematosus and rheumatoid arthritis
Source: Lupus Sci Med. 2018 Sep 25;5(1):e000278. doi: 10.1136/lupus-2018-000278 (PMC6173266; doi:10.1136/lupus-2018-000278)
Supplement: Supplementary data [file lupus-2018-000278supp002.docx]

**Table S1. Specificities of auto-antibodies.**

Class I HLA Class II HLA

*SLE Nulliparous Males*

A031282 B*3801 DRB*0402, DQA*0301/DQB*0302

A001178 DRB*0301

A002779 DRB*0301

A031729 DRB*0801

*SLE Nulliparous Females*

A024137 A*0201, B*0801, B*5701, C*0602

A001370 DRB*0801

A013185 DQA*0201/DQB*0202

A031215 DRB*0401

A031763 DRB*0801

*SLE Parous Females*

A038326 C*0602, C*0702

A031194 B*5001

A071590 B*3701

A000202 C*0102, C*0602 DRB*1501

A009490 C*0702

A062252 C*0602

A001169 DRB*0301

A002782 DRB*0301

A010373 DRB*1501, DQA*0102/DQB*0602

A014339 DRB*0401

A016440 DRB*0801

A024234 DRB*0404

A038256 DRB*0301, DRB*0801

A069396 DRB*0401

A071421 DRB*0301

A073546 DRB*0301,

*RA Parous Females*

A071538 B*0801

A027042 DRB*0301

A059556 DRB*0401

A028038 DRB*0801, DRB*1101

A065891 DRB*0404

A053190 DRB*0401

A062506 DRB*0301
